# Metal hypersensitivity in patient with posterior lumbar spine fusion: a case report and its literature review

**DOI:** 10.1186/1471-2474-15-314

**Published:** 2014-09-26

**Authors:** Xianping Shang, Ling Wang, Depeng Kou, Xunyuan Jia, Xianglong Yang, Meng Zhang, Yilong Tang, Pengrui Wang, Shijin Wang, Yan Xu, Hong Wang

**Affiliations:** Department of Spine Surgery, First Affiliated Hospital of Dalian Medical University, Dalian, 116011 People’s Republic of China; Department of Oncology, First Affiliated Hospital of Dalian Medical University, Dalian, People’s Republic of China

**Keywords:** Lumbar spine, Decompression with fusion, Aseptic loosening, Prostheses and implants/adverse effects

## Abstract

**Background:**

Metal hypersensitivity, mostly documented in prosthesis implantation, is a rare complication after arthroplasty. Such cases become rarer and more difficult to diagnose when it comes to lumbar surgery.

**Case presentation:**

We present the case of a 52-year-old female patient with reoccured low back pain and sciatica after posterior lumbar decompression and fusion (PLDF) for her lumbar disc herniation. The initial clinical and radiological examinations showed no pathologies. Further imaging and histopathological studies in later period revealed an aseptic loosening of the hardware and an aseptic inflammatory response which was diagnosed to be metal hypersensitivity. To our knowledge, few allergic cases in the matter of spinal fusion were reported so far.

**Conclusions:**

Metal hypersensitivity after spinal fusion should be considered in patients with representation of postoperative back pain. And elaborate history taking would conduce a lot to it’s diagnose.

**Electronic supplementary material:**

The online version of this article (doi:10.1186/1471-2474-15-314) contains supplementary material, which is available to authorized users.

## Background

Patients can be sensitive to metal debris released from the hardware for orthopedics treatment, and presenting with pain, swelling, inflammatory skin reactions, implant loosening, and fistula formation. Metal hypersensitivity, most documented among total joint arhtroplasty, is one of the rare complications after orthopedic procedures, with small number of cases after spine arthrodesis. The typical clinical presentation towards the high level of metal ions after surgery is persistent unexplained pain, or a development of unexplained pain after an initial pain-free interval[1], usually within the first 6 months after implantation. The level of metal ion in serum decrease rapidly with time after revision but still remained above normal levels for 4 years after surgery[2].

In patients representing late-onset persistent pain and trouble walking, after spinal fusion, an infection or incomplete surgery must be considered, but also the possibility of metal hypersensitivity. Once an infection and imcomplete surgery have been excluded, metal hypersensitivity is about to come into notice. As we know, all metals which are in contact with biologic systems are subject to corrosion. The released ions could activate the immune system by forming metal-protein complexes, considered as candidate antigens to elicite hypersensitivity responses[3].

In this report, we presented a case of 52-year-old female with metal hypersensitivity in a few months after Posterior Lumbar Decompression and Fusion (PLDF) due to lumbar intervertebral disc herniation, aiming to highlight the uncertainty in the diagnostic process and the significance of a complete history taking.

This report was made under the ethical approval of The Medical Ethic Committee of the First Affiliated Hospital of Dalian Medical University.

## Case presentation

**Figure 1:**
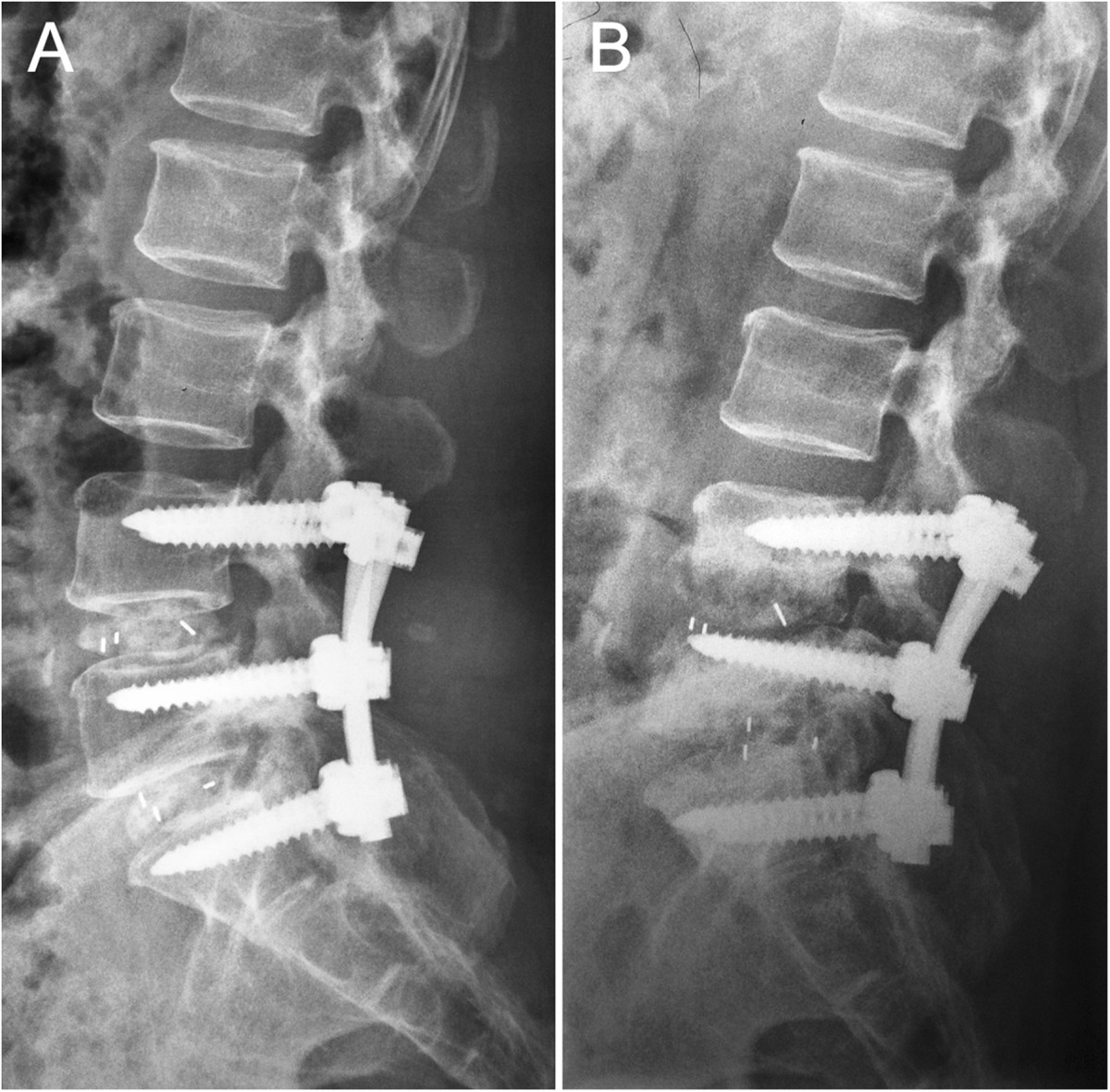
**Lateral radiographs of the internal fixator and spine. (A)** An immediate postoperative lateral radiograph. **(B)** Radiograph at 3 mo after postoperative, showing areas of osteolysis and malposition of the pedicle screws.

**Figure 2:**
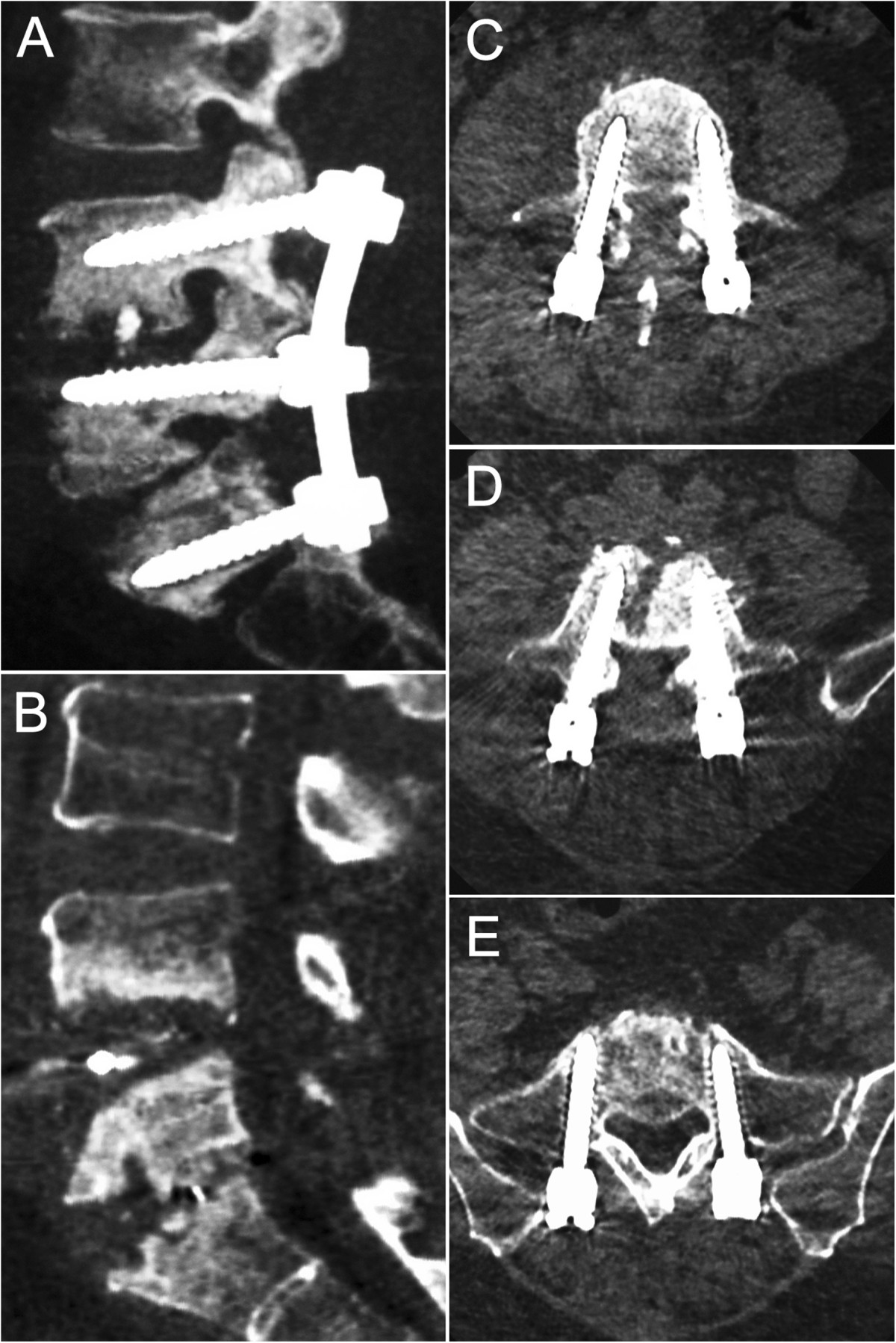
**Osteolysis between L4 to S1.** Sagital, Computed tomography (CT) revealing osteolysis between L4 to S1 **(A, B)**. Note the gap around the pedicle screws in the vertebrae body(especially in **C** and **E**), and attenuation of the erector spinae is also evident in the axial view **(C, D, E)**.

A 52-year-old female patient, sustained low back pain for 1 year with complaints of numbness in both lower extremities, underwent PLDF (Medtronic Sofamor Danek USA, Inc) bilaterally from L4 to S1 in September 2011 and got instant relief. 3 month postoperatively, however, the patient represented with low back pain. The increasingly severly deep aching pain had intensified over the next 5 months and ultimately gave rise to trouble walking accompanied by a mild sphincter disturbances before she’s review. This had been thought to be related to delayed postoperative infection. But, the patient had no fever and her physical examination was unremarkable other than a slightly swelling with tenderness over the operative region. Neither a high skin temperature nor flare was found. On the contrary, the incision scar had met a criterion of primary healing. Serial blood analysis showed erythrocyte sedimentation rate (ESR), C-reactive protein (CRP), and complete blood count with differential (CBC w/ diff) were within normal limits. X-ray films showed the slightly shifted internal fixators partially lost its function (Figure 1), and computed tomography (CT) gave evidence of loosening and osteolysis (Figures 2 and3). Findings of magnetic resonance imaging (MRI) of the operative lumbar spine revealed topical swelling of soft tissue around the prosthesis and cloud sign of the adipose layer (Figure 4). There was no diagnostic explanation for her pain. None of the clinic findings supported delayed postoperative infection. As no other cause for the low back pain (commonly named failed back surgery syndrome) could be identified, a quick decision was made to proceed with removal of the pedicle screw system.

**Figure 3:**
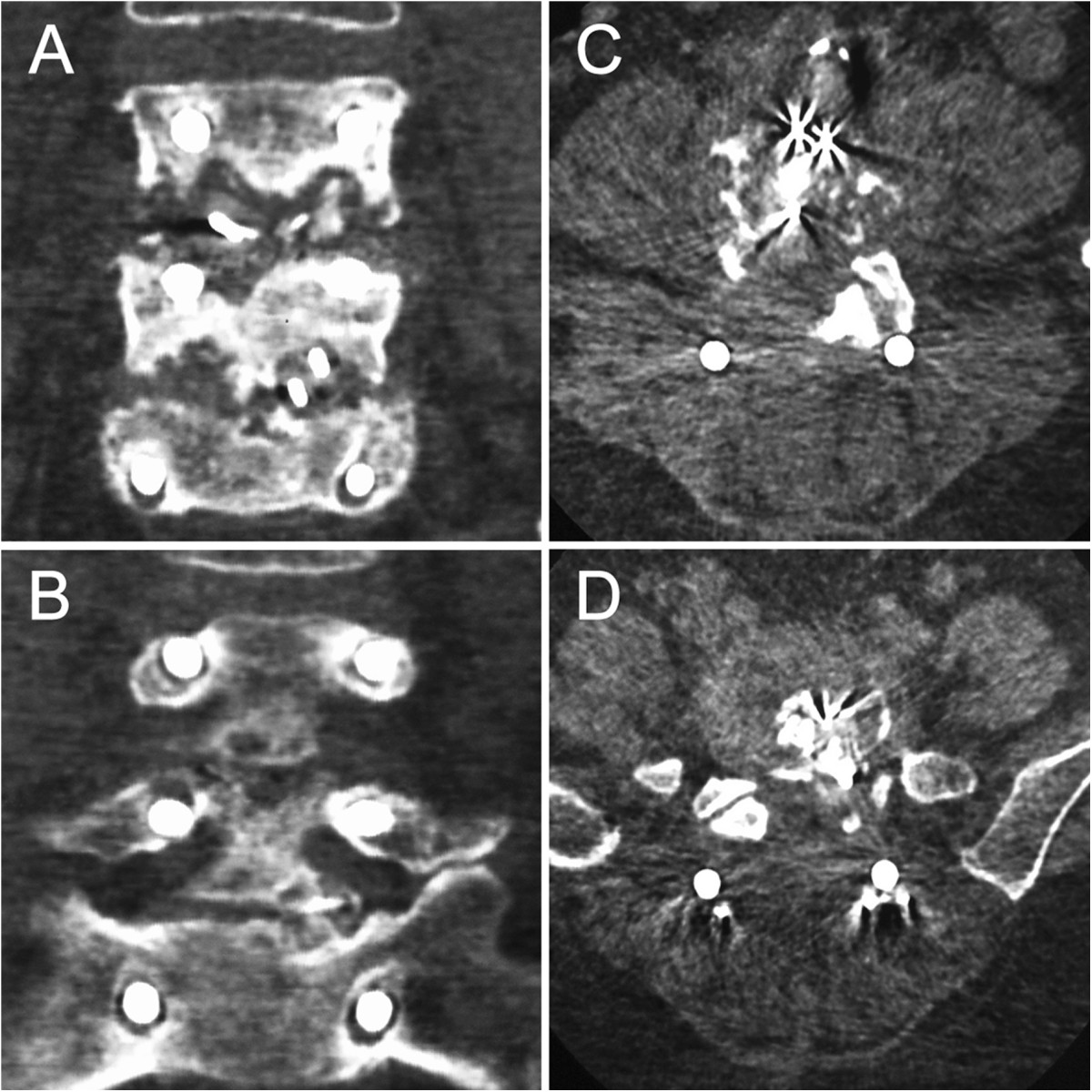
**Musculoskeletal and fixator changes.** Coronal, Computed tomography (CT) showing loosening of the pedicle screws and the osteolysis **(A, B)**. Axial view indicating metallosis in the intervertebral space, the erector spinae with lower attenuation is also identified **(C, D)**.

**Figure 4:**
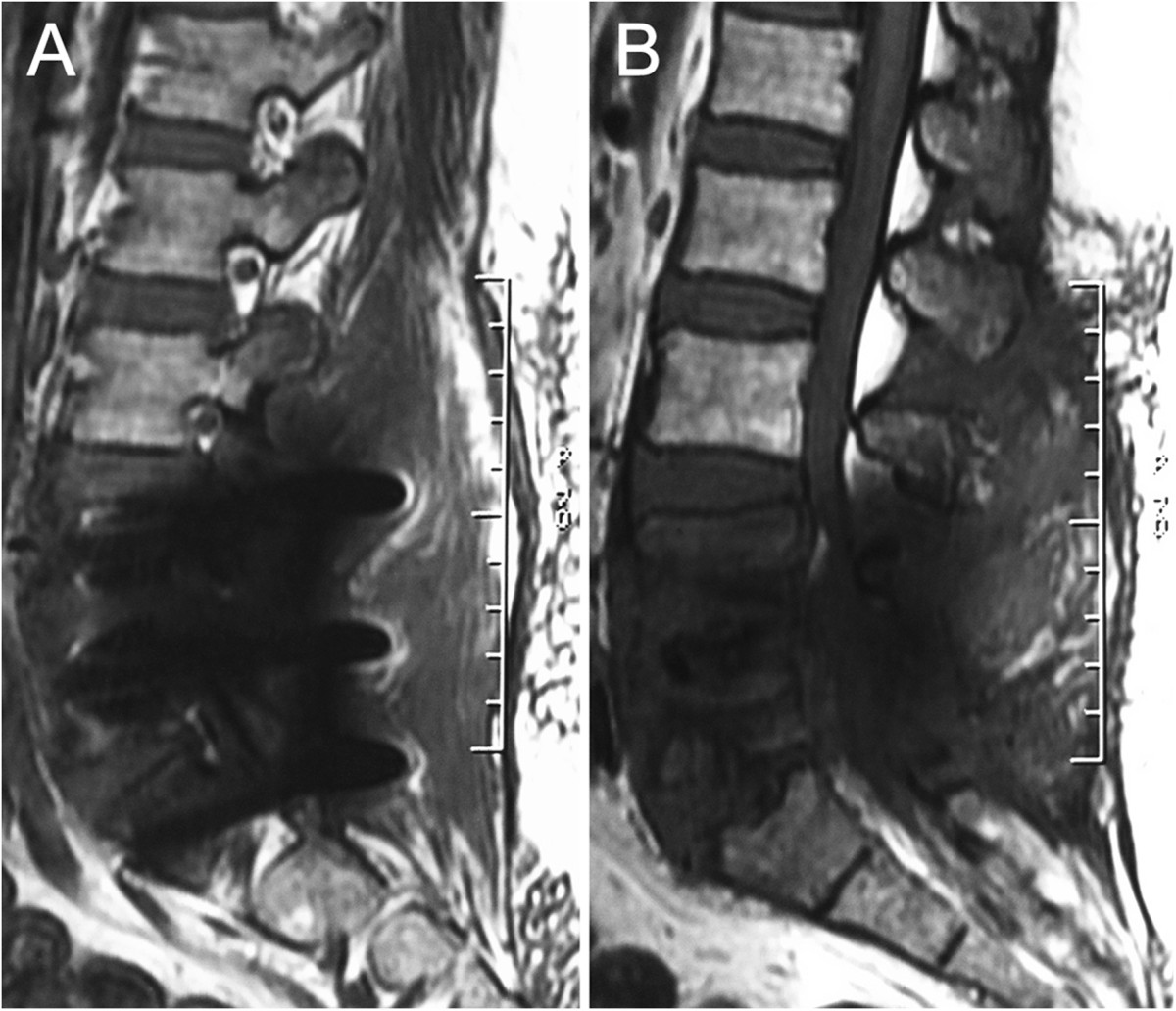
**Sagital, 3.0 T, T1-weighted magnetic resonance image(MRI) demonstrate the swelling adjacent soft tissue (A) and “cloud sign” in the adipose layer (B).**

**Figure 5:**
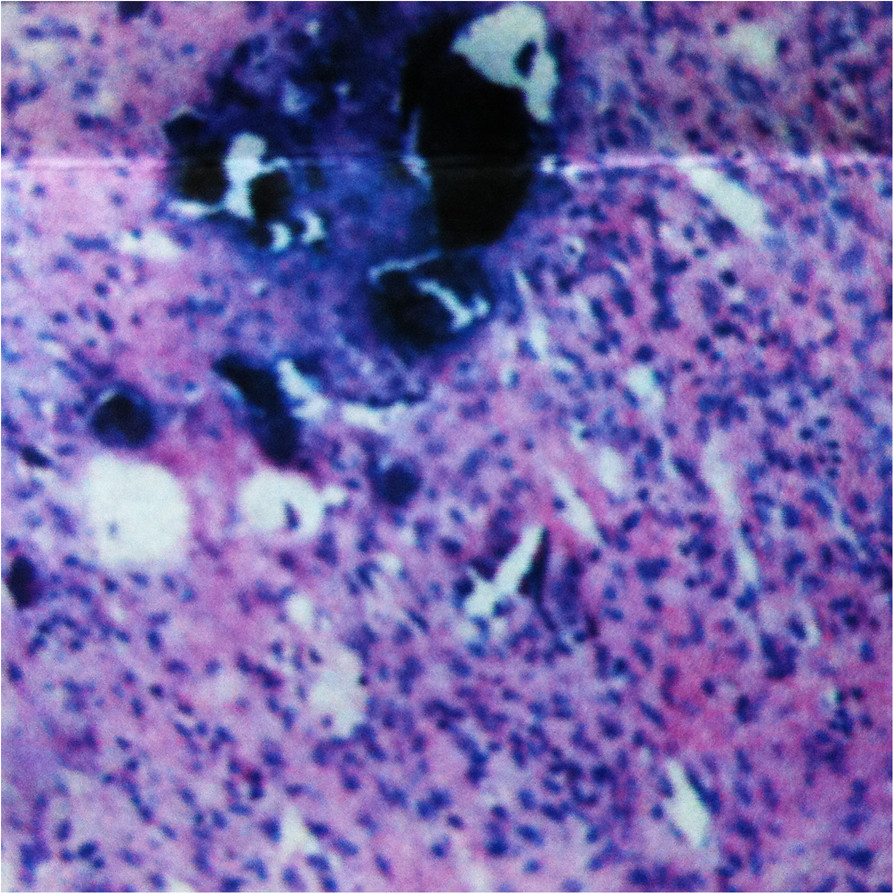
**Histopathology (10 × 20) of resected periprosthetic specimen revealed infiltration of massive lymphocytes.**

During surgery, the 6 pedicle screws were found to be very loose off the vertebrae and effortlessly removable, resulting to lost their fixation functon. No pus, caseous necrosis or tumor was found over the periprosthetic tissue. But a small granulation tissue was identified around the pedicle scerw in the L4/5 level and sent to biopsy along with a piece of bone from the L5/S1 intervertebral space. A predominance infiltration of lymphocytes with massive fibroblasts and neocapillaries was found in this specimen (Figure 5), yet no evidence of infection. We still offered a postoperative treatment toward infection, including intravenous antibiotics application for 3 weeks and continuous antibiotic lavage and drainage for 25 days. Bacteria was not verified from the drainage culture, yet. The removal of pedicle screw system alleviated all the symptoms, especially the back pain, reduced to a lower level but persisted for a while.

She gave a clear history of skin sensitivity to metal for many years before receiving PLDF and was unable to wear a metal watch or ring. After PLDF, she did not notice rashes or irritation over the low back area, or any other skin reaction, and other clinical evidence of infection.

## Conclusions

Metal hypersensitivity as a complication after arthroplasty is rare[4], however, it is likely that cases involving implant-related metal sensitivity have been underreported because of the difficulty of diagnosis[5, 6]. Even so, prospective studies have shown a higher incidence of metal hypersensitivity in patients with implant failure[7].

Metal hypersensitivity has been associated with arthroplasty recipients mainly with metal on metal bearing surfaces. This could be attributed to the immunologic effects and /or cell toxicity mediated by exposure to wear debris[8–10]. It is hypothesized that metal particles are slowly released from the prosthetic bearing surfaces as a by-product of normal wear. And these particles are subject to corrosion resulting in producing high levels of ions potentially causing cell death. Wear debris from metal prosthetics are demonstrable in adjacent periprosthetic soft tissue as well in distant sites such as lymph nodes, liver, and spleen[11–13]. Adjacent tissue reactions to the debris have been given a variety of names: aseptic lymphocytic-vasculitis-associated lesions (ALVAL), pseudotumor, necrosis, adverse reaction to metal debris, and adverse local tissue response, with or without a clear underlying cause. But it’s generally believed that these wear particles, in conjunction with native proteins, form haptens that elicit a late onset type IV hypersensitivity in the local tissue[2, 3].

Most alloys used for orthopaedic implants rely on an oxidative layer to protect them from corrosion. Mechanical factors such as applied stress, fretting, and even micromotion will cause this protective layer to break down. Besides crevices formed during the manipulation also will defect the protective layer[14]. The corrosive process accelerated, once the alloy was exposed to the chemical factors from body fluid through the imperfection of the oxidative layer. With corrosion, metal ions are released into the body. Spine fixators are static load-bearing devices subjected to micromotion and fretting at least until a successful fusion would have been achieved. However, even after a successful arthrodesis, there is continued load sharing between the implants and the fusion mass and this may lead to continued stress within the implants, potentially resulting in fretting corrosion. Therefore, spine implants can cause metal ion and debris release from fretting corrosion with elevating levels in body fluids, especially in the periprosthetic tissue. This has been reported by several studies about metal ion levels in spinal implants[14–16]. Moreover, titanium particulate debris at the level of a spinal arthrodesis could elicit a cytokine-mediated particulate-induced response favoring pro-inflammatory infiltrates and increased expression of intracellular tumor necrosis factor-alpha, increased osteoclastic activity, and cellular apoptosis in an animal model[17]. In the clinical setting, the presence of titanium particulate, secondary to corrosion of spinal implants could serve as the impetus for both late-onset inflammatory-infectious complications and long-term osteolysis in the established posterolateral fusion mass[17].

Levels of metal ions measured in blood after spinal arthrodesis is comparable to that seen after total joint arthroplasty[14]. Metal concentrations in serum was not seemed as a useful indicator of hardware loosening or implant failure[16], but may be associate with the systematic reaction, including urticaria, eczema, and pruritus. As to the topo-reaction around the periprosthetic tissue, metal deposition in the surrounding tissue seems to be attributable and can be a local destructive response leading to pain, eczematous reaction, ostoslysis, and loosening of the implants.

Patients presenting a late-onset postoperative pain with no clinical evidence of infection stand a good chance of metal hypersensitivity and a further evaluation should be performed. Basic physical examination, blood tests including ESR and CRP, radiographs, preoperative and intraoperative biopsy may be helpful to make a definite diagnosis.

There is no practical guide in the literature on how to differentiate between metal hypersensitivity and infection in a painful spinal fusion. Consequently, the evaluation largely depends on the process of the pain elimination. Routine laboratory data including the ESR and CRP are noted to have a high diagnostic accuracy towards infection[18]. Studies also give confirmable sensitivities and specificities of various tests to diagnose infection. For example the combination of white cell sulphur colloid scan and Technetium Tc99m bone scan with a high accuracy in diagnosing infection was used in diagnosing metal hypersensitivity related to implants failure has also been reported[19].

Although culture of intraoperative specimens, including tissue samples and swabs is an effective method to identify infection, however, this could present false positives or false negatives due to cross contamination or previous antimicrobial therapy.

Biopsy study is reported to be the most accurate predictor of infection, featuring a predominant infiltration of neutrophils in periprosthetic tissues, while the characteristic histologic appearance of hypersensitivity reactions to metal prostheses is dominated by lymphoplasmacytic infiltration and droplike inclusions in the cytoplasm of macrophages[20]. The appearance in a biopsy specimen may help the surgeon to reach a definite diagnosis. It has been recommended that in cases of suspected metal hypersensitivity, an arthroscopic biopsy followed by histological analysis should be performed[19].

Assessment of hypersensitivity has historically been conducted in vivo by skin patch testing and in vitro by leukocyte migration inhibition testing (LMIT). Patch testing has been used for many years with a greater frequency of positivity in patients with metal implants[21], but recommendations for routine testing are still controversial[3, 18, 22, 23]. The short duration of dermal contact in skin patch testing is different to the long term closed environment of the orthopaedic implant, and the relationship between dermal and deep implant sensitivity is yet unknown. Concerns also exist that patch testing could possibly induce hypersensitivity in a previously insensitive patient. In vitro tests like lymphpcyte transformation testing, LMIT appear more promising but are expensive and labor intensive. Besides, these tests are unavailable in many hospitals and have not been proven in the clinical setting.

The primary symptom in our case is delayed aggravating postoperative back pain, that is, there was a pain free/alleviation interval, which was consistent with most metal hypersensitivity cases. We note that the back pain before removing the hardware was severe and persistent without postural relief even when she stayed in bed. However after removal of the hardware, the pain alleviated greatly when stay in bed, not that much when she got out of bed. That is due to the instability of lumbar spine, resulted from endplates destruction and laminectomy performed during primary surgery. We also believe that the length of this pain free/ alleviation interval is negatively related to the integriy of protective layer on the hardware surface. Unlike severe cases, her pain was limited to the low back region, no other cutaneous or vascular symptoms were found over the whole body. This seems optimistic because a small amount of metal debris was produced in the short segment applied hardware and only localized in the surrounding area. Another prominent symptom, herein, is urinary retention occurred 8 months after the primary surgery. The urinary retention is consistent with Cauda Equina Syndrome (CES), which is a well-known complication for lumbar disc herniation. But the episode of CES after a complete decompressive procedure is quite unusual. In total disc replacement cases, Guyer RD et al. found the matellosis soft-tissue were responsible for CES[24]. However, no abnormal tissue was found close enough to cause CES in our case.

Metal hypersensitivity, usually requiring complete removal or revision of the metal instruments, could be an unpleasant incident to both patients and surgeons, and is often neglected due to its low incidence and unpredictable occurance. Moreover, it also gives a diagnostic challenge because of the lack of practical diagnosis guide. Since it’s a well-recognized causative factor for reoccurrence of persistent preoperative pain after spinal fusion, precaution and diagnosis of this entity should always keep in mind. Although preoperative history-taking alone appears to be insufficient for identifying patients with metal sensitivity[7], it is still strongly recommended to be taken thoroughly, because this could provide a strong clue towards the delayed postoperative pain in time, and therewith, a timely customized examination and treatment could be conducted to benefit the patient tremendously. In this case, the patient sustained low back pain for another 5 months because of the difficulty in diagnosis, but the related signs and symptoms resolved shortly after removal of the metal instruments like other cases reported[25–27].

### Consent

Written informed consent was obtained from the patient for publication of this Case report and any accompanying images. A copy of the written consent is available for review by the Editor of this journal.

## Authors’ original submitted files for images

Below are the links to the authors’ original submitted files for images. Authors’ original file for figure 1 Authors’ original file for figure 2 Authors’ original file for figure 3 Authors’ original file for figure 4 Authors’ original file for figure 5
