# Supplementary figures and images for: Metal hypersensitivity in patient with posterior lumbar spine fusion: a case report and its literature review
Source: BMC Musculoskelet Disord. 2014 Sep 26;15:314. doi: 10.1186/1471-2474-15-314 (PMC4192797; doi:10.1186/1471-2474-15-314)

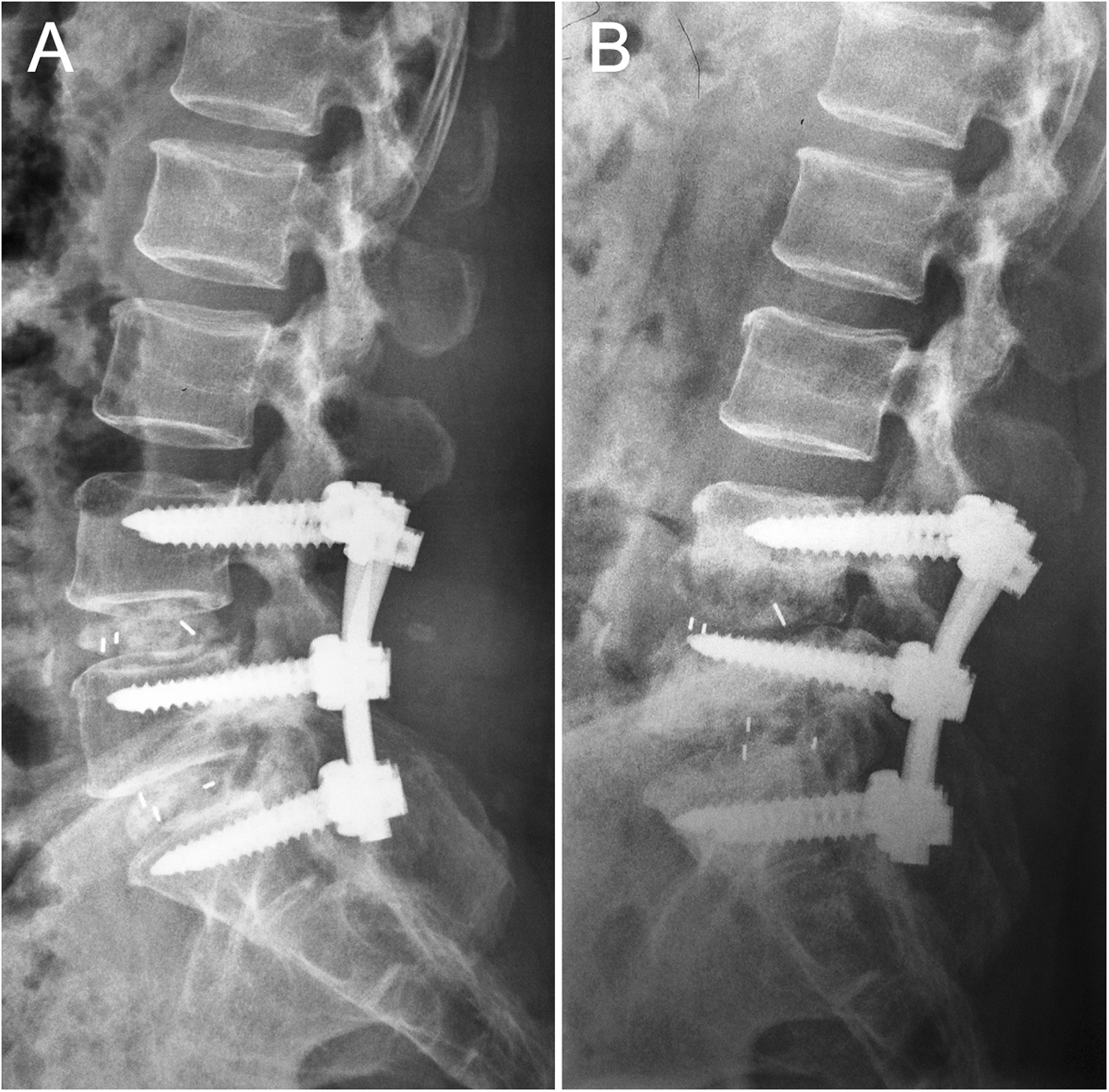

Supplement: Supplementary file 1 — Authors’ original file for figure 1 [file 12891_2014_2264_MOESM1_ESM.tif]

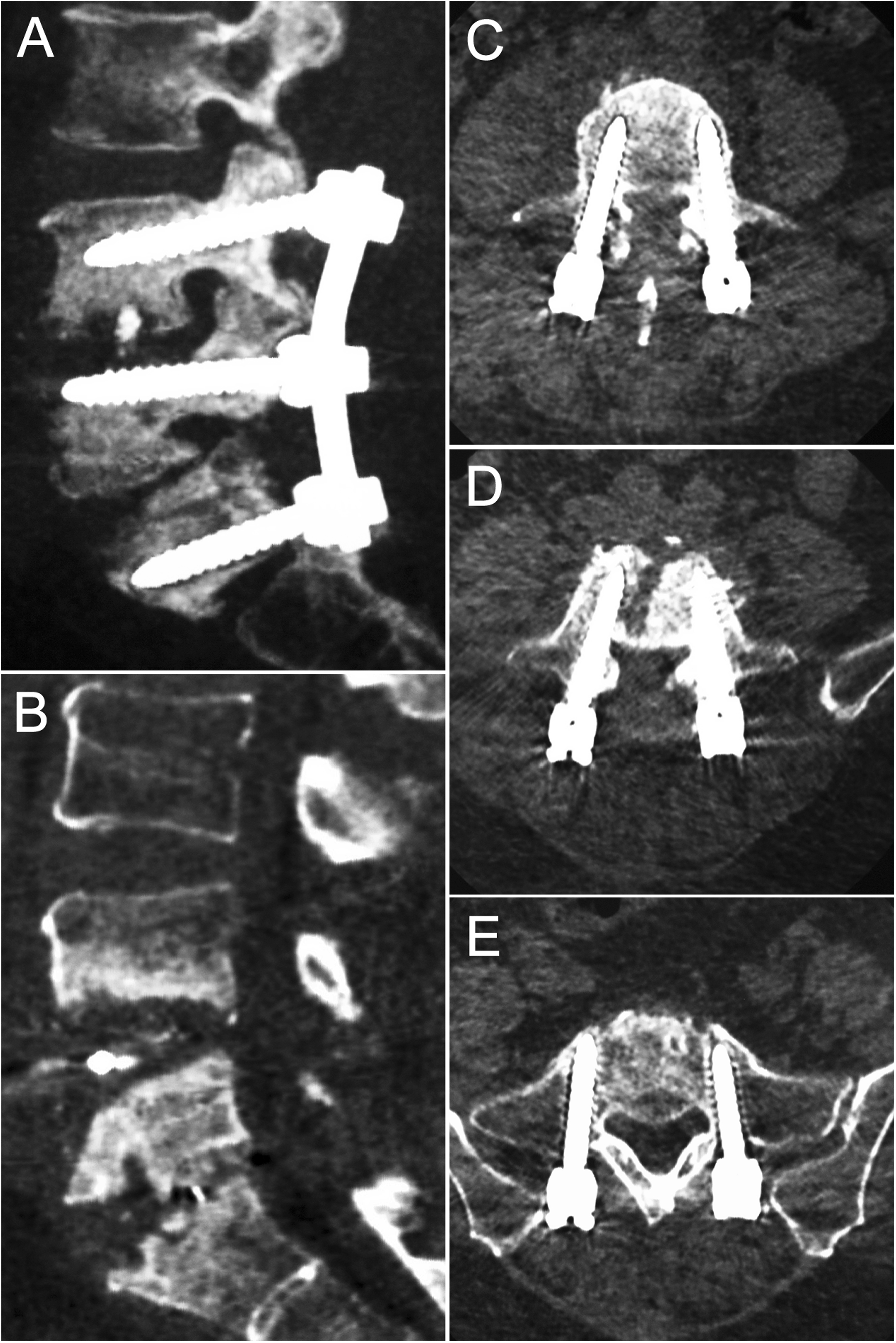

Supplement: Supplementary file 2 — Authors’ original file for figure 2 [file 12891_2014_2264_MOESM2_ESM.tif]

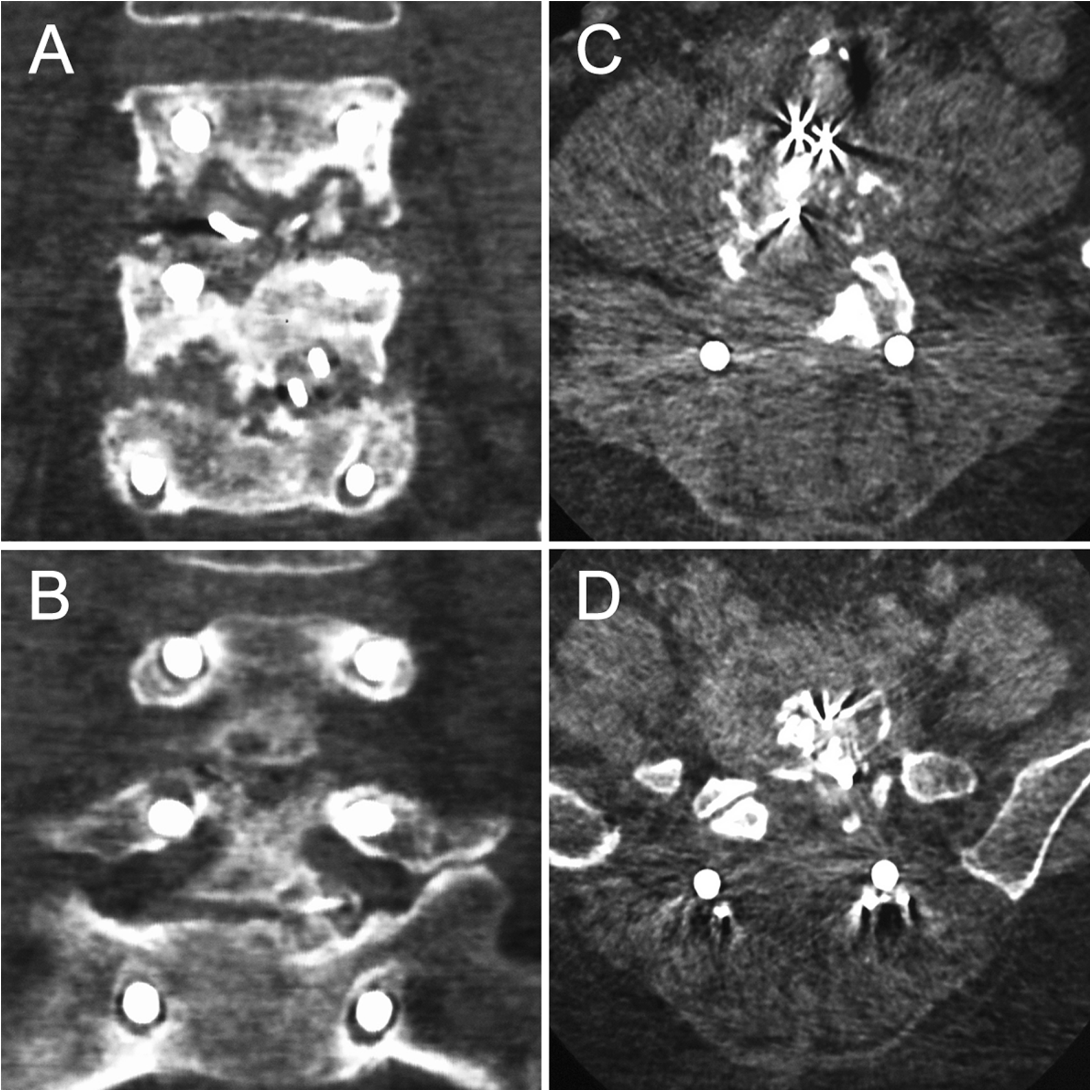

Supplement: Supplementary file 3 — Authors’ original file for figure 3 [file 12891_2014_2264_MOESM3_ESM.tif]

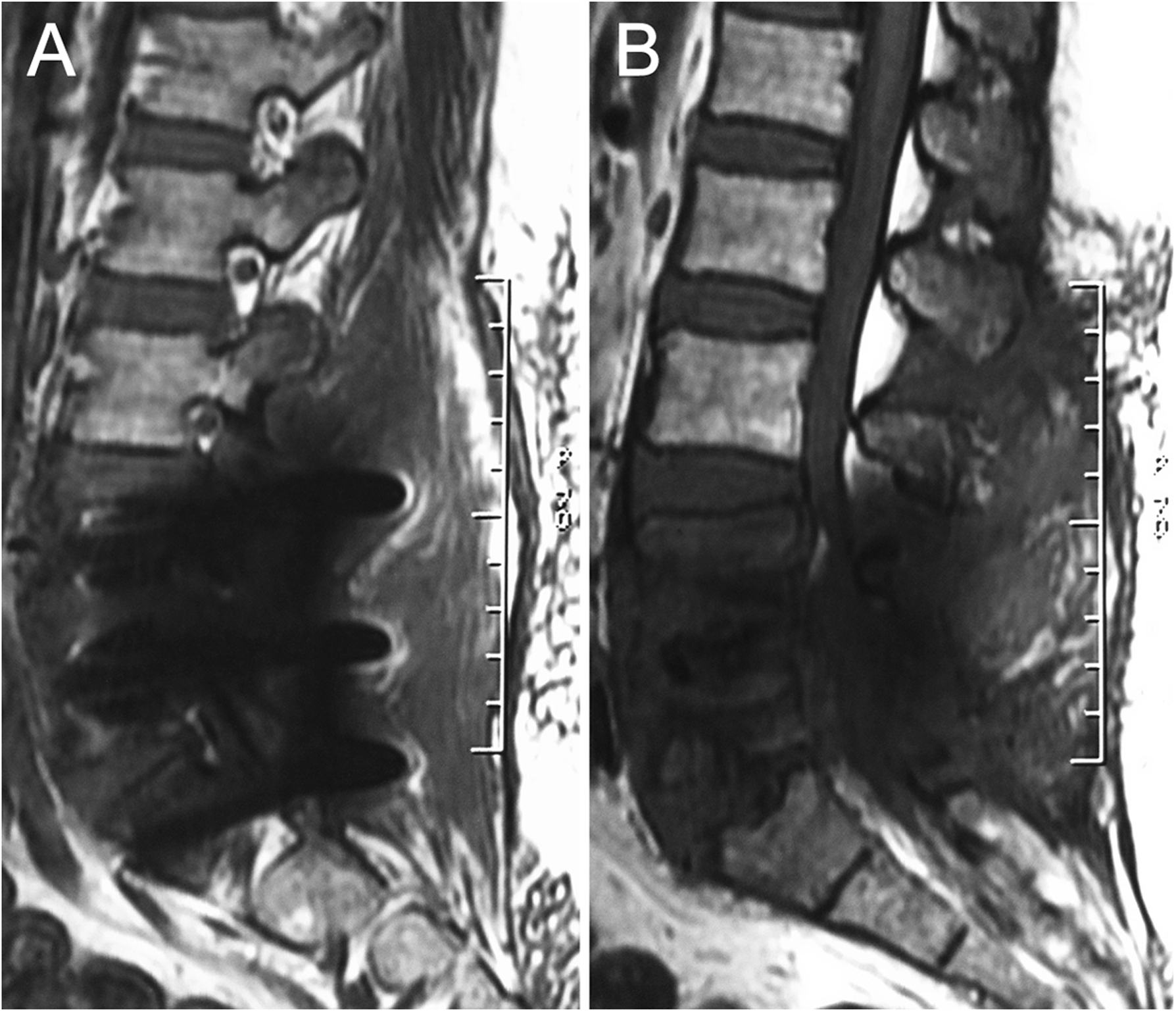

Supplement: Supplementary file 4 — Authors’ original file for figure 4 [file 12891_2014_2264_MOESM4_ESM.tif]

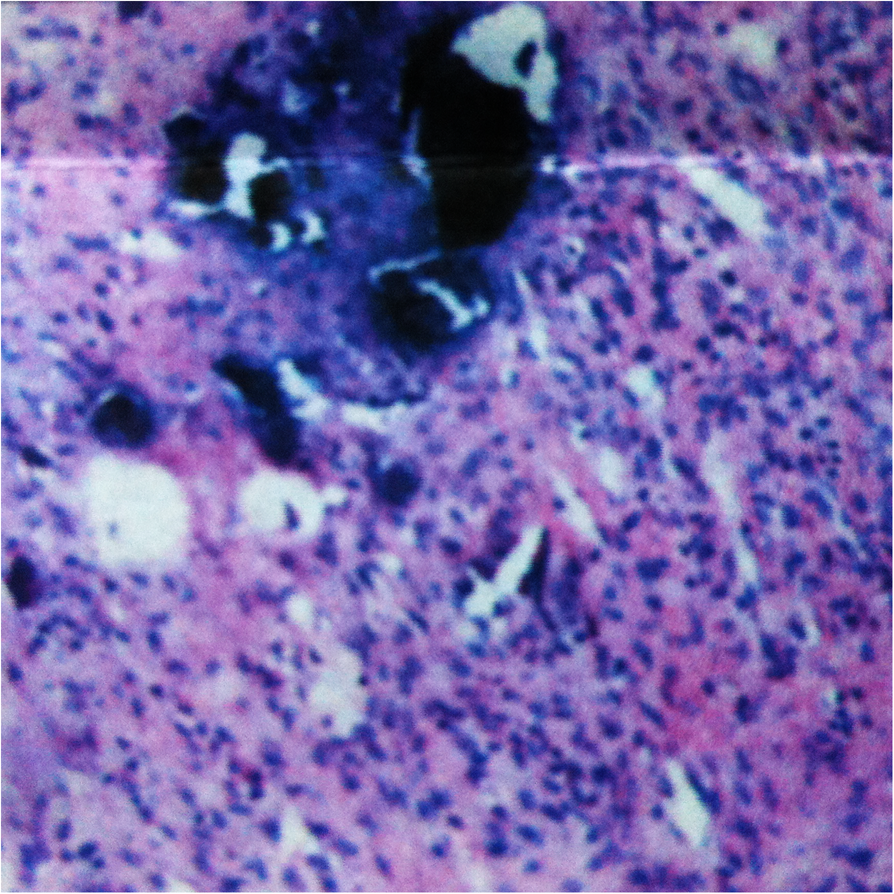

Supplement: Supplementary file 5 — Authors’ original file for figure 5 [file 12891_2014_2264_MOESM5_ESM.tif]
